# Integrating experimental data and mechanistic modeling to assess potential lead exposure from tampon use

**DOI:** 10.1093/toxsci/kfag052

**Published:** 2026-05-07

**Authors:** Corie A Ellison, Patrick R Doyle, Christina A Haven, Denise M McClenathan, Cindy M Obringer, Kara E Woeller

**Affiliations:** Global Product Stewardship, The Procter & Gamble Company, Mason, OH 45040, United States; Corporate Functions Analytical, The Procter & Gamble Company, Mason, OH 45040, United States; Corporate Functions Analytical, The Procter & Gamble Company, Mason, OH 45040, United States; Corporate Functions Analytical, The Procter & Gamble Company, Mason, OH 45040, United States; Global Product Stewardship, The Procter & Gamble Company, Mason, OH 45040, United States; Global Product Stewardship, The Procter & Gamble Company, Cincinnati, OH 45232, United States

**Keywords:** tampons, plasma protein binding, metals, mechanistic modeling, exposure refinement

## Abstract

Trace levels of lead (Pb) have been reported in tampons, prompting concerns about potential exposure during menstrual product use. However, the presence of a chemical in a product does not necessarily translate to biologically relevant exposure. In this study, we characterized the distribution and binding of Pb in menstrual fluid and developed a deterministic, compartmental mass-balance model to evaluate the release and fate of Pb that may be present as an inadvertent trace impurity in tampons. Our experimental measurements demonstrated that Pb preferentially partitions to the red blood cell (RBC) fraction of menstrual fluid. The remaining Pb was distributed in the plasma fraction, where the majority was protein-bound. These distribution and binding characteristics were broadly comparable to those reported for systemic blood. Utilizing these data, a mechanistic model was parameterized to describe the release of Pb from a tampon into menstrual fluid, partitioning between menstrual fluid compartments, reabsorption into the tampon during fluid uptake, and potential permeation across vaginal tissue. Under the conservative assumptions evaluated here, model simulations for a 4-h tampon wear scenario predicted that the majority of theoretically released Pb is reabsorbed into the tampon, with only a very small fraction (<1 ng; <0.3%) available for potential absorption into vaginal tissue. Sensitivity and alternative release scenario analyses demonstrated that predicted tissue uptake remained minimal across various plausible conditions. Collectively, these findings underscore the importance of integrating chemical presence data with physiological context and mechanistic modeling to inform exposure assessment and support science-based evaluation of product safety.

Tampons are a widely used menstrual hygiene product designed to absorb menstrual fluid and provide comfort and convenience during menstruation. Made predominantly of cotton, rayon, or a combination of both, tampons are inserted into the vagina, where they absorb menstrual flow (Hochwalt et al. 2023). Their design allows for discreet use and facilitates physical activity that might be uncomfortable or impractical with other menstrual products (Omar et al. 1998). The mental and physical health benefits of tampon use are well recognized (Cardoso et al. 2021). Psychologically, tampons can enhance quality of life by allowing greater freedom and mobility, reducing anxiety associated with leaks during physical activities, and contributing to a sense of normalcy and control during menstruation (Brooks-Gunn and Ruble 1982). Physically, tampons effectively manage menstrual flow, promote hygiene, reduce odor, and may be more comfortable for some users compared with pads (Choi et al. 2021).

In August 2024, Shearston et al. (2024) evaluated the concentration of 16 metals in multiple tampon brands and reported measurable concentrations of all metals assessed. The authors concluded that tampon use may be a potential source of metal exposure, although the study did not evaluate whether the metals were released by the product or absorbed by the user. In response, the U.S. Food and Drug Administration (FDA) conducted an independent systematic literature review to investigate potential contaminants in tampons (US FDA 2024), ultimately reaffirming the historical safety of tampons. The American College of Medical Toxicology, in response to Shearston et al. (2024), expressed concern that the paper could lead to unwarranted health worries or the use of dangerous therapies (Parris et al. 2025), a sentiment echoed by Öberg (2024). Despite this consensus, certain groups have questioned the safety of tampons, highlighting studies that identify the presence of contaminants. These inquiries often rely on harsh, non-physiological conditions to extract contaminants from products, and findings are often presented without adequately contextualizing them within human health risk assessments. Testing guidelines, such as the ISO 10993 medical device standards (subchapter 18 specifically), employ stringent extraction conditions and the use of aggressive solvents, recommending initial testing under extreme conditions to characterize the device. Such methods often exaggerate the amount of extractable material and do not necessarily reflect realistic human exposure scenarios.

To accurately gauge reasonable potential exposure to any chemical within a tampon, several considerations must be acknowledged. One must evaluate the physiological conditions under which chemicals may leach from tampons, assess the fraction of chemicals that remains bound and bio-inaccessible, and consider the extent to which a chemical may penetrate the vaginal epithelium. The menstrual cycle is a complex physiological process characterized by hormonal fluctuations that prepare the body for potential pregnancy. Menstrual fluid, the byproduct of this cycle, is composed of blood, cervical mucus, vaginal secretions, and endometrial tissue. Tampons function by absorbing this fluid, effectively managing menstrual flow and promoting hygiene. Menstrual fluid may provide a unique matrix that has a high potential to bind various chemicals, including environmental contaminants. As tampons absorb this fluid, they may also trap these chemicals, influencing their bioavailability and potential systemic absorption and exposure.

In the current study, we investigate the distribution and binding characteristics of lead (Pb) in menstrual fluid. We chose Pb as a model contaminant due to its well-characterized toxicological profile and known behavior in systemic blood. We hypothesize that Pb distribution and binding in menstrual fluid will resemble those in systemic blood, with predictable differences arising from differences in matrix composition. Although systemic blood primarily consists of water, intact red blood cells (RBCs), and proteins such as albumin, menstrual fluid additionally contains tissue debris and lysed cells, which may alter the behavior of Pb. In systemic blood, lead predominantly binds to hemoglobin within RBCs, as well as other proteins, with a small fraction found in plasma (Ong and Lee 1980; deSilva 1981; Sugawara et al. 1990; Schütz et al. 1996). As with other xenobiotics, the unbound fraction of Pb is relevant for toxicological assessments, as it represents the bioavailable form that can potentially induce toxic effects (Bergdahl et al. 2006; Poulin and Arnett 2017).

In this study, we characterize the distribution of Pb within menstrual fluid, quantify the fraction of Pb that remains unbound in this matrix, and compare these properties with those observed in systemic blood and plasma. In addition, we develop a deterministic, compartmental mass-balance model to describe the hypothetical release of a chemical from a tampon into menstrual fluid and its subsequent fate within the vaginal lumen. This modeling framework is then applied to evaluate the fate of Pb that may be present as an inadvertent trace impurity in tampons if it were released under typical conditions of use.

## Materials and methods

### Chemicals

Lead (II) acetate trihydrate, (CAS number: 6080-56-4) was > 99% pure and received from Sigma Aldrich (Catalog #215902, Lot#MKCV2064). Dulbecco’s phosphate-buffered saline with Ca++ and MG++ (DPBS+) was utilized as the receiver chamber buffer.

### Biological fluids

Menstrual fluid was collected noninvasively from adult donors using a menstrual cup. Women who self-identified as current cup users were provided instructions to collect menstrual fluid over a 24-h period on designated days of the menstrual cycle (Day 1, 2, 3, and/or 4). Donors documented the time of each collection (i.e. cup insertion and removal), dispensing collected fluid into Hemogard 4 ml sodium heparinized tubes from BD Vacutainer at the end of each collection period. Donors labeled each tube sequentially in the order in which it was collected and then refrigerated the sample. At the end of a 24-h period, all vials were transferred into a cooler with ice packs to maintain refrigeration during transport to the testing site. The process of sample collection was considered minimal risk, and an Institutional Review Board was deemed unnecessary. The study was conducted in the spirit of compliance with the good clinical practice regulations. Study participants provided written informed consent.

Pooled (5 female and 5 male donors) human plasma isolated from systemic blood was purchased from BioIVT.

### Hematocrit measurement of menstrual fluid

The percentage of RBCs was measured for each menstrual fluid sample using an Autocrit Ultra 3. The menstrual fluid sample was centrifuged at the pre-programmed speed for 5 min, and the RBCs were visually quantified against the internal hematocrit scale.

### Distribution of endogenous and exogenous Pb in menstrual fluid

Endogenous Pb (i.e. Pb that enters menstrual fluid via systemic circulation) content was measured in each whole menstrual fluid sample, as well as the plasma and RBC fractions isolated from menstrual fluid. Plasma and RBC fractions were isolated from menstrual fluid by centrifugation of whole menstrual fluid at 4,100 × g for 10 min.

To evaluate the distribution of exogenous Pb (i.e. Pb added directly to menstrual fluid), the whole menstrual fluid samples were dosed with 1, 10, or 25 ng Pb/ml in metal-free plastic conical tubes. Lead(II) acetate trihydrate was initially dissolved in water at a concentration of 1 mg Pb/ml. Spiking solutions were then prepared by diluting the 1 mg/ml stock solution in water to 100× of each test concentration. The 100× spiking solutions were then added to the menstrual fluid and inverted to ensure thorough mixing. The volume of the spiking solution constituted 1% of the total assay volume and is not anticipated to impact the overall osmolarity of the sample. Spiked samples were rocked for 10, 60, or 240 min in a 37 °C incubator. Following incubation, the Pb concentration was measured in the whole menstrual fluid, plasma fraction isolated from menstrual fluid, and RBC fraction isolated from menstrual fluid. Any extra menstrual fluid was centrifuged to separate the fractions, and the plasma fraction was removed and frozen at −20 °C for future use.

### Binding of exogenous Pb in plasma isolated from menstrual fluid or systemic blood

Plasma protein binding of Pb was measured using the rapid equilibrium dialysis plates with a molecular weight cutoff of 8,000 Da. Plasma isolated from menstrual fluid or systemic blood was spiked with 1, 10, or 25 ng Pb/ml and aliquoted to the donor chambers. DPBS+ served as the receiver chamber buffer. The sealed plate was placed on an orbital shaker at 100 rpm in a 37 °C incubator for 4 h to allow the Pb to come to equilibrium in the test system. At the conclusion of the incubation, each chamber was sampled for analysis of Pb content.

### Measurement of Pb in biological fluids by ICP-MS

#### Test sample preparation—total microwave digestion

Samples (0.2–0.5 g) were placed into pre-cleaned 7 ml TFM UltraWAVE vials (Milestone, Bergamo, Italy) and 2 ml of nitric acid was added to each vial. Samples were then digested using an UltraWAVE Microwave Digestion System, heating to a final temperature of 240 °C. After digestion, samples were transferred to pre-cleaned 15 ml polypropylene tubes, and then 200 µl of 1 µg/ml Iridium (Ir) was added to each tube as an internal standard before diluting to 10 ml with deionized water. The resulting test solutions were visually checked to confirm full digestion of the sample, such that the solution yielded a clear solution with no fine particulates present. The test solutions were analyzed by ICP-MS for Pb.

#### Fortified sample preparation

Matrix spikes were prepared for each analysis following the same procedure as the samples, except adding the appropriate aliquot of Pb standard prior to microwave digestion. Spike levels varied depending on the expected sample concentration. Typical spike levels were 15, 25, 150 , 500, and 5,000 pg/ml in the test solution. These concentrations correspond to 0.75 ng/g, 1.25 ng/g, 7.5 ng/g, 25 ng/g, and 250 ng/g for a 0.2 g sample.

#### Standard preparation

Working standards were prepared by combining appropriately diluted reference standards (Inorganic Ventures, Christiansburg, Virginia), covering minimally 5 to 1,000 pg/ml in 20% v/v nitric acid with 20 ng/ml Ir as the internal standard. A mid-range calibration standard or various other calibration standards were also used as a QC standard to monitor drift throughout the analysis. An additional standard was prepared from a second lot/source of lead reference standard (SCP Science) to verify the validity of the calibration curve.

#### ICP-MS analysis

Prepared standards and test samples were analyzed using ICP-MS (Agilent 7900 ICP-MS, Agilent Technologies, Inc., Santa Clara, California) for the determination of Pb. The instrument was optimized according to manufacturer recommendations, and analysis was conducted via summation of *m*/*z* 206, *m*/*z* 207, and *m*/*z* 208 in no gas mode. This summation is used to ensure accuracy for Pb quantitation, as its isotopic composition can vary considerably in nature. Method performance was assessed in each analysis through suitability requirements, including residual error limits for each calibration standard (≤25% for the lowest standard and ≤20% for all other standards), and requirements for recovery of QC standards and fortified samples (80% to 120%). Concentration of the lead acetate spiking solutions was verified through ICP-MS to ensure appropriate dosing solution preparation.

### Data analysis

#### Adjusting for hematocrit content

All of the reported concentrations of Pb in plasma and RBC fractions of menstrual fluid were adjusted for hematocrit levels, as this allows for a more accurate representation of the Pb concentrations relative to the proportion of intact RBCs in the samples, facilitating meaningful comparisons between the components of menstrual fluid. Hematocrit content specific to a given donor, menstrual cycle month, and menstrual cycle day was used for the correction. Adjustments for hematocrit content were done as follows:


Concentration blood=Concentration plasma × (1-hematocrit fraction)

Concentration blood=Concentration RBC × hematocrit fraction

#### Calculating the percent of exogenous Pb unbound in plasma isolated from menstrual fluid or systemic blood

The concentration of Pb in the receiver chamber (DPBS+) is free (F) while the Pb in the donor chamber (plasma) is a mix of free and bound (F + B) Pb at the end of the incubation. The free and bound Pb are in equilibrium with each other in the donor chamber, whereas the free Pb in both the donor chamber and the receiver chamber are also in equilibrium with each other. The percentage unbound is calculated as [F/F + B)] × 100.

#### Calculating partition coefficients

Partition coefficients (K) were calculated using the approach of Hinderling (1997) and utilized the concentration of Pb in the menstrual fluid-derived RBC and plasma. The partition coefficient for menstrual RBC-to-menstrual plasma (*K*_M_rbc:M_plasma_) was calculated as:


$$K_{M\_\mathrm{rbc}:M\_\mathrm{plasma}}=\mathrm{concentration}\;\mathrm{in}\;\mathrm{menstrual}\;\mathrm{fluid}\;\mathrm{derived}\;\mathrm{RBC}/\mathrm{concentration}\;\mathrm{in}\;\mathrm{menstrual}\;\mathrm{fluid}\;\mathrm{derived}\;\mathrm{plasma}$$

Note that *K*_M_rbc:M_plasma_ is independent of hematocrit and does not utilize hematocrit-adjusted blood concentrations. The partition coefficient for menstrual fluid-to-menstrual plasma (*K*_M_fluid:M_plasma_) was calculated as:


$$K_{M\_\mathrm{fluid}:M\_\mathrm{plasma}}=K_{M\_\mathrm{rbc}:M\_\mathrm{plasma}}\;*\;\mathrm{fraction}\;\mathrm{menstrual}\;\mathrm{fluid}\;\mathrm{hematocrit}+(1-\mathrm{fraction}\;\mathrm{menstrual}\;\mathrm{fluid}\;\mathrm{hematocrit})$$

### Mass-balance model describing the release of a chemical from a tampon into menstrual fluid

#### Description of model

**Fig. 1.**
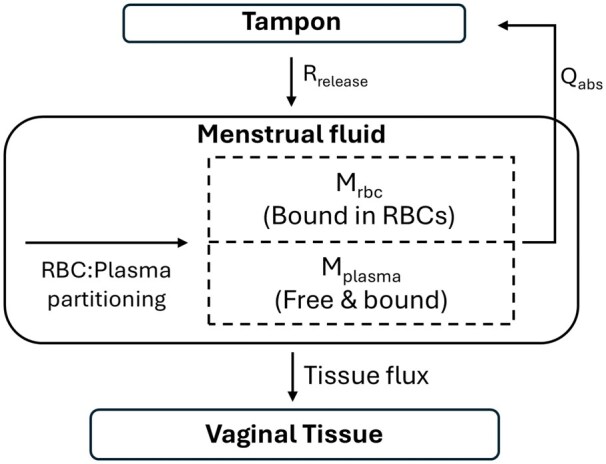
Schematic of mass-balance model describing release of a chemical from a tampon into menstrual fluid and its subsequent fate within the vaginal lumen.

A deterministic, compartmental mass-balance model was developed to describe the hypothetical release of a chemical from a tampon into menstrual fluid and its subsequent fate within the vaginal lumen (Fig. 1). The model explicitly represents chemical partitioning within menstrual fluid, reabsorption into the tampon with fluid uptake, and permeation across vaginal tissue. The model was implemented and solved using Berkeley Madonna (University of California, Berkeley). The model consists of 4 mass-balance compartments: (i) menstrual fluid–derived plasma, (ii) menstrual fluid–derived RBCs, (iii) vaginal tissue (as a cumulative uptake compartment), and (iv) tampon reabsorption (cumulative). Chemical release from the tampon into menstrual fluid was modeled as a constant rate (ng/h). Upon release, chemical mass is distributed between menstrual fluid plasma and RBC fractions. Menstrual fluid absorption into the tampon was represented as a volumetric flow (ml/h). Chemical mass dissolved in menstrual fluid plasma and RBC fractions was removed proportionally with fluid uptake, resulting in reabsorption of the chemical into the tampon. Plasma-associated and RBC-associated chemicals are tracked separately to maintain mass balance. Only the unbound fraction of the chemical in menstrual plasma was assumed available for permeation across vaginal tissue. Tissue uptake was modeled using a permeability-area product approach, with the apparent permeability coefficient (Papp, cm/s) and exposed tissue surface area (cm^2^). Permeation was treated as a unidirectional sink, and absorbed mass was accumulated in a tissue compartment. The code for the model is available in the Supplementary Information.

#### Application of the model for Pb

The mass-balance model was parameterized to describe the release and subsequent fate of Pb that may be present in a tampon as a trace impurity. Model parameter values were obtained from a combination of experimental measurements generated in the current study, published literature sources, and internal data, as summarized in Table 8. The rate of Pb release from the tampon into menstrual fluid was estimated to be 66 ng/h. This release rate was derived using published data reporting Pb concentrations in tampons following harsh, unrealistic total digestion analytical methodology, for which a geometric mean concentration of 120 ng Pb per gram of tampon material was reported (Shearston et al. 2024). Although we believe this value is exaggerated compared with values obtained from more physiologically relevant methodology, it was used as a provisional value which is beyond a ‘worst-case’ scenario. Assuming a representative tampon mass of 2.2 g, this corresponds to a total Pb content of approximately 264 ng per tampon. For the baseline simulation, the total Pb content was assumed to be released continuously and uniformly over the duration of tampon wear, which was set to 4 h. The assumed tampon mass of 2.2 g is representative of a commonly used “Regular” absorbency tampon and was based on internal data. Experimental data generated in the current work were used to parameterize the distribution of Pb between the plasma and RBC fractions of menstrual fluid, as well as the fraction of Pb that remains unbound in plasma isolated from menstrual fluid. These experimentally derived partitioning values were incorporated directly into the model. Menstrual fluid absorption into the tampon was modeled as a volumetric uptake rate of 1 ml/h, based on data reported by Levin and Wagner (1986), Atsuko et al. (2024) and supported by internal data. The volume of free fluid present in the vaginal lumen was set to 0.5 ml, consistent with published estimates (Mitchell et al. 2011). The vaginal tissue surface area available for chemical absorption was set to 21 cm^2^, representing the approximate surface area of vaginal tissue in direct contact with a fully saturated tampon, as estimated from internal data. Although the total vaginal epithelial surface area has been reported to be approximately 90 cm^2^ (Li et al. 2013), this value overestimates the surface area based on our understanding of physical contact limitations, bypass fluid dynamics, and user behavior and product use. Therefore, only the tissue in close proximity to the tampon was considered relevant for chemical transfer in the present model. In the absence of permeability data for Pb through vaginal tissue, Caco-2 permeability data were utilized as a proxy. The permeability coefficient (Papp) of Pb across vaginal tissue was assumed to be on the order of 10^−6 ^cm/s. Bolan et al. (2021) reported Pb permeability values ranging from 0.13 × 10^−6^ to 0.17 × 10^−6 ^cm/s across a Caco-2 cell monolayer. The Caco-2 permeability assay was conducted under specific experimental conditions: 21 d post-seeding of the cells, at pH 7.4, with apical-to-basolateral transport, a temperature of 37 °C, and continuous agitation (70 Hz plate shaker) to minimize the unstirred water layer. Because Caco-2 permeability data have not been extensively validated as a surrogate for vaginal epithelial transport, a conservative approach was adopted by increasing the permeability estimate by one order of magnitude. Accordingly, a Papp value of 1 × 10^−6 ^cm/s was used in the model simulations. All simulations were conducted over a 4-h duration, corresponding to the typical wear time for a tampon.

Local sensitivity analysis was performed to evaluate the relative influence of each parameter in the model on cumulative tissue uptake. Parameters were evaluated individually, and the rank order of importance was determined based on the calculated sensitivity coefficients. The sensitivity coefficient was calculated as previously described (Yoon et al. 2009) and is defined as the percent change in the dose metric of interest (cumulative tissue uptake) resulting from a change in a particular parameter.


$$\mathrm{Sensitivity}\;\mathrm{Coefficient}=\frac{(a-b)/b}{(c-d)/d}$$

where *a* is the dose metric following modification of a parameter value, *b* is the dose metric with the original parameter value, *c* is the modified parameter value, and *d* is the original parameter value. A sensitivity coefficient close to zero indicates that a parameter has little influence on the dose metric, whereas a sensitivity coefficient of 1 indicates a 1:1 relationship between a parameter and the dose metric (e.g. a 1% change in the parameter input value results in a 1% change in the dose metric). A positive sensitivity coefficient indicates an increase in the dose metric with an increase in the parameter value. A negative sensitivity coefficient implies that the parameter influences the dose metric in an inverse direction.

All model parameters were subject to the sensitivity analysis, and a 1% change in parameter value relative to baseline was done for a 4-h simulation exposure, utilizing a value of 66 ng/h for the release rate of Pb from a tampon. This percent of change in parameter value was utilized to ensure the model stayed in the region of biological plausibility for the parameter. Each model parameter was categorized as having low, medium or high influence on cumulative tissue uptake using previously reported criteria (IPCS 2010):

- Low: 0.1 ≤ sensitivity coefficient < 0.2
- Medium: 0.2 ≤ sensitivity coefficient < 0.5
- High: 0.5 ≤ sensitivity coefficient

## Results

### Menstrual fluid sample collection and characteristics

Menstrual fluid was collected from a group of donors, spanning the reproductive age range [22 to 51 yr of age (median 28)] (Table 1). The volume of menstrual fluid provided by the donors varied significantly and influenced the quantity of analyses we conducted on each sample. Specifically, some donors contributed sufficient menstrual fluid, enabling us to perform all or multiple studies outlined in the methods section. In contrast, other donors provided limited volumes, restricting our analyses to the measurement of endogenous Pb (i.e. Pb that enters menstrual fluid via internal systemic circulation) in menstrual fluid. Physical characteristics (presence of clots and mucus, thickness, stringiness) of the menstrual samples were visibly different; however, no measurements were taken to quantify these characteristics. Whole menstrual fluid samples retained their integrity when stored in the refrigerator for a short duration (approximately 24 to 48 h), but then became increasingly viscous with notable color change over time and were discarded.

**Table 1.** Demographics of menstrual fluid donors.

Donor ID	Age	Race
RP54	51	Caucasian
RP83	46	Caucasian
RP115	22	Caucasian
RN1	28	Caucasian
RN2	25	Caucasian
RN3	24	Caucasian
RN4	26	Caucasian/Hispanic/Latinx
RN5	33	Caucasian
RN6	36	Black/African American/Caucasian
RN8	26	Caucasian
RN9	27	Caucasian
RN10	30	Caucasian

Upon processing the menstrual fluid samples through centrifugation, we observed distinct separation into 3 main layers. The uppermost layer was considered menstrual fluid-derived plasma, characterized by its relative clarity and fluidity. The middle layer was considered the buffy coat, which contains a thin layer of white blood cells and platelets. The bottom layer was considered the menstrual fluid-derived RBCs, which settled to the base of the centrifuge tube. Notably, the coloration of the plasma isolated from menstrual fluid differed visibly from that of plasma isolated from systemic blood. Although systemic blood-derived plasma typically has an amber hue, the plasma isolated from menstrual fluid displayed a more pronounced reddish tint. We hypothesize that this color difference is attributable to the presence of lysed RBCs, which release hemoglobin and contribute to the color observed in the menstrual fluid-derived plasma. Additionally, the tinge in the menstrual fluid-derived plasma may also result from components of the shedding endometrial tissue, further distinguishing it from systemic blood-derived plasma. The color of the menstrual fluid-derived plasma differed across the donors.

### Hematocrit measurements of menstrual fluid

Hematocrit measurements serve as an indicator of intact RBCs present in a blood sample. Understanding hematocrit content is particularly relevant to the current work, as it allows for the normalization of measurements and the determination of partition coefficients for Pb within menstrual fluid. We attempted to obtain hematocrit readings for all collected menstrual fluid samples. However, in one instance, the sample was so thick that it would not flow into the capillary tube. In another case, we encountered difficulties in obtaining readings due to the samples bursting the capillary tubes. We attributed this issue to the high viscosity and density of these specific menstrual fluid samples (small viscous volume, many blood clots, as well as other solid debris), which exerted excessive outward pressure on the capillary tubes during the measurement process. These samples (donors RP115 and RN3) were used for the determination of endogenous Pb concentration in menstrual fluid, but were not used in any of the other additional analyses.

Our analysis included hematocrit measurements conducted on menstrual fluid across 4 d within a single menstrual cycle, across 3 different menstrual cycles, and involving multiple donors (Table 2). For donor RP54, hematocrit values remained relatively consistent throughout the 4 d of sampling, with values ranging from 20% to 27%. On day 2 of the menstrual cycle, we collected menstrual fluid samples across 3 different months from 2 donors, RP54 and RP83. The hematocrit content across these 3 mo exhibited variability of 11% for donor RP54 and 27% for donor RP83. The average hematocrit on day 2 was found to be 25% for RP54 and 37% for RP83, indicating differences in hematocrit levels between the 2 donors. When examining hematocrit levels of menstrual fluid collected on day 2 across multiple donors (*N* = 10), the average hematocrit was 25%, with a standard deviation of ±7%.

**Table 2.** Hematocrit measurements for menstrual fluid.

Study purpose	Donor	Cycle day	Cycle month	Hematocrit (%)
Day-to-day	RP54	1	–	27
		2		25
		3		20
		4		24
Month-to-month	RP54	2	1	30
			2	25
			3	19
	RP83	2	1	52
			2	25
			3	35
Donor-to-donor	RP54	2	–	25^a^
	RP83			37^a^
	RP115			Failed^b^
	RN1			25
	RN2			27
	RN3			Failed^b^
	RN4			20
	RN5			24
	RN6			25
	RN8			36
	RN9			16
	RN10			17
	Average			24

a Average across 3 menstrual cycles.

b Assay failed due to high viscosity of sample.

### Endogenous concentration of Pb in menstrual fluid

To establish a baseline understanding of the presence of Pb in menstrual fluid, we first measured the concentration of endogenous Pb in whole menstrual fluid samples collected using menstrual cups. In this context, “endogenous Pb” refers to Pb that enters menstrual fluid via the systemic circulation. Additionally, we assessed the distribution of this endogenous Pb concentration to determine whether Pb was primarily located in the plasma or RBC fraction of menstrual fluid. This analysis was conducted over 4 d within a given menstrual cycle and specifically on day 2 across 3 different cycles. Furthermore, we examined day 2 samples from multiple donors (Table 3).

**Table 3.** Endogenous concentration of Pb in menstrual fluid and isolated fractions.

Study purpose	Donor	Cycle day	Cycle month	Whole menstrual fluid (average ± SD)	Menstrual plasma (average ± SD)	Menstrual RBCs (average ± SD)	Number of replicates^a^
Day-to-day	RP54	1	–	3.30 ± 0.05	0.24 ± 0.19	2.66 ± 0.22	3,3,3
		2		3.01 ± 0.08	0.33 ± 0.23	2.64 ± 0.04	3,3,3
		3		2.43	0.55	1.18	1,1,1
		4		3.47	0.13	1.71	2,2,2
Month-to-month	RP54	2	1	3.44	0.07	2.83	2,2,2
			2	3.01 ± 0.08	0.33 ± 0.23	2.64 ± 0.04	3,3,3
			3	2.74 ± 0.09	0.10 ± 0.02	1.52	3,3,2
	RP83	2	1	2.57	0.16	2.16	2,2,2
			2	2.29 ± 0.23	0.74 ± 0.10	0.99 ± 0.20	3,3,3
			3	2.66 ± 0.05	0.79 ± 0.09	1.85 ± 0.06	3,3,3
Donor-to-donor	RP54	2	–	3.01 ± 0.3	0.18 ± 0.18	2.37 ± 0.59	8,8,7
	RP83			2.50 ± 0.22	0.61 ± 0.29	1.61 ± 0.54	8,8,8
	RP115			1.96			2,0,0
	RN1			4.63 ± 0.18	0.23 ± 0.01	1.29	3,3,2
	RN2			2.28 ± 0.14	0.11 ± 0.01	1.91 ± 0.03	3,3,3
	RN3			4.08			2,0,0
	RN4			1.94	0.37 ± 0.02	1.42	2,3,2
	RN5			0.98 ± 0.47	0.29 ± 0.11	0.68 ± 0.17	3,3,3
	RN6			2.28 ± 0.52	0.92 ± 0.03	0.69 ± 0.13	3,3,3
	RN8			4.27 ± 0.11	0.45	2.47	3,2,2
	RN9			4.81	0.96	1.44	1,1,1
	Average			2.98 ± 1.28	0.47 ± 0.31	1.46 ± 0.55	

a Number of replicates corresponding to measurement in whole menstrual fluid, menstrual plasma and menstrual RBCs, respectively. RP54 and RP83 data spans 3 menstrual cycles.

The concentrations of Pb reported for plasma and RBC fractions of menstrual fluid were adjusted for hematocrit levels, as this allows for a more accurate representation of the Pb concentrations relative to the proportion of intact RBCs in the samples, facilitating meaningful comparisons between the components of menstrual fluid. Our findings indicated that the endogenous concentration of Pb in whole menstrual fluid remained consistent across the 4 d within a given cycle, with values consistently within 1 ppb of each other. The hematocrit-corrected concentration of Pb in menstrual fluid-derived plasma ranged from 0.13 to 0.55 ppb, which was consistently lower than the concentration found in menstrual fluid-derived RBCs, which ranged from 1.71 to 2.66 ppb. Similarly, for day 2 samples collected across 3 different cycles, the concentration of Pb in whole menstrual fluid was stable. For donors RP83 and RP54, the endogenous Pb values were relatively constant, remaining within 1 ppb of their respective monthly samples. Again, the concentration of Pb in menstrual fluid-derived plasma was consistently lower than that found in menstrual fluid-derived RBCs. To further investigate the endogenous concentration of Pb in menstrual fluid, we assessed an additional 9 donors. The average endogenous concentration of Pb in whole menstrual fluid ranged from 0.98 to 4.81 ppb across the donors, and the group average was 2.98 ppb. Within this group, the average endogenous Pb concentration in menstrual fluid-derived plasma was 0.47 ppb, whereas the average concentration in menstrual fluid-derived RBCs was 1.46 ppb.

### Time course and dose response of exogenous Pb in menstrual fluid

To investigate the dynamics of exogenous Pb distribution within menstrual fluid, we conducted time course studies involving incubations of 10, 60, and 240 min. These experiments aimed to understand the rate at which exogenous Pb, introduced at a concentration of 10 ng/ml, partitions between the plasma and RBC fractions of menstrual fluid (Table 4). For donor RP83, we observed that the concentrations of Pb in the plasma and RBC fractions of menstrual fluid were on the same order of magnitude across the 3 time points measured. Notably, there was minimal variation in the average concentration of Pb across these time points, suggesting a relatively stable partitioning behavior of Pb between these 2 components of menstrual fluid. For donor RP54, a similar trend was noted regarding the Pb concentration in menstrual fluid-derived RBCs, which remained consistent across the different time points. The concentration of Pb in menstrual fluid-derived plasma exhibited an apparent decrease over time; however, some of this variation may be attributed to the relatively low concentrations being measured (0.2 to 1.2 ppb). Based on these data, we selected an incubation duration of 60 min for subsequent studies.

**Table 4.** Time course for distribution of exogenous Pb (10 ng/ml) added to day 2 menstrual fluid.

Donor	Incubation time (min)	Whole menstrual fluid (average ± SD)	Menstrual plasma (average ± SD)	Menstrual RBCs (average ± SD)	Number of replicates^a^
RP54	10	11.30 ± 1.33	1.24 ± 0.31	7.33 ± 0.74	6,6,6
	60	13.44 ± 3.28	0.38 ± 0.23	11.03 ± 2.73	9,9,8
	240	12.71 ± 0.12	0.19 ± 0.01	9.55 ± 1.08	3,3,3
RP83	10	10.90 ± 4.63	6.50 ± 3.96	4.04 ± 1.71	8,8,8
	60	11.16 ± 4.76	5.69 ± 3.54	4.93 ± 2.18	8,8,8
	240	13.52 ± 0.03	4.38 ± 0.03	7.66 ± 0.70	3,3,3

a Number of replicates corresponding to measurement in whole menstrual fluid, menstrual plasma and menstrual RBCs, respectively.

Dose-response studies were conducted to study the impact of exogenous Pb concentration on the distribution behavior between menstrual fluid plasma and RBC fractions. Specifically, doses of 1, 10, and 25 ng/ml of Pb were incubated for 60 min in whole menstrual fluid samples prior to fractionation into menstrual fluid-derived plasma and RBC components (Table 5). We observed distinct distribution patterns among the donors. For donor RP83, the concentration of Pb was almost equally distributed between the plasma and RBC fractions isolated from whole menstrual fluid. In contrast, donor RP54 exhibited a markedly different behavior, with approximately 10 to 30 times more Pb concentrated in the menstrual fluid-derived RBC fraction compared with the plasma fraction. To further investigate these binding dynamics, menstrual fluid samples from 6 additional donors were dosed with Pb at a concentration of 10 ng/ml and incubated for 60 min before fractionation. The distribution of Pb into either menstrual fluid-derived RBCs or plasma varied across these donors, likely reflecting the inherent interindividual variability in menstrual fluid composition. The distribution profile for the 6 additional donors more closely resembled the distribution pattern of RP83 than that of RP54.

**Table 5.** Distribution of endogenous Pb (0 ng/ml) and exogenous Pb (1, 10, 25 ng/ml) added to day 2 menstrual fluid for 60 min.

Donor	Pb dose (ng/ml)^a^	Whole menstrual fluid (average ± SD)	Menstrual plasma (average ± SD)	Menstrual RBCs (average ± SD)	Number of replicates^b^
RP54	0	3.01 ± 0.3	0.18 ± 0.18	2.37 ± 0.59	8,8,7
	1	4.71 ± 0.54	0.36 ± 0.1	3.55 ± 0.16	3,3,3
	10	13.44 ± 3.28	0.38 ± 0.23	11.03 ± 2.73	9,9,8
	25	27.73 ± 3.64	0.73 ± 0.54	17.01 ± 2.62	5,6,5
RP83	0	2.5 ± 0.22	0.61 ± 0.29	1.61 ± 0.54	8,8,8
	1	3.48 ± 0.64	1.13 ± 0.47	1.87 ± 0.39	8,8,8
	10	11.16 ± 4.76	5.69 ± 3.54	4.93 ± 2.18	8,8,8
	25	29.2 ± 8.08	13.52 ± 9.51	12.79 ± 6.07	8,8,8
RN1	10	15.17 ± 0.12	6.41 ± 0.02	6.36 ± 0.002	3,3,3
RN2	10	13.81 ± 0.10	4.80 ± 0.01	6.26 ± 0.02	3,3,3
RN4	10	14.40 ± 0.44	5.02 ± 0.04	5.50 ± 0.16	3,3,3
RN5	10	10.21 ± 0.16	4.93 ± 0.50	3.62 ± 0.98	3,3,3
RN6	10	11.90 ± 0.03	7.49 ± 0.03	4.12 ± 0.28	3,3,3
RN8	10	12.41	13.25	6.41	2,2,2

a Exogenous Pb (1, 10 or 25 ng/ml) was dosed into day 2 menstrual fluid and incubated for 60 min. Pb dose of 0 ng/ml represents background exposure.

b Number of replicates corresponding to measurement in whole menstrual fluid, menstrual plasma and menstrual RBCs, respectively.

### Partition coefficients

With measurements of hematocrit and the concentrations of Pb in whole menstrual fluid, menstrual fluid-derived RBCs, and menstrual fluid-derived plasma, we were able to calculate partition coefficients (*K*) for Menstrual RBC-to-Menstrual plasma (*K*_M_rbc:M_plasma_) and Menstrual fluid-to-Menstrual plasma (*K*_M_fluid: M_plasma_). These coefficients provide insight into the distribution dynamics of Pb within the menstrual fluid under varying conditions. Partition coefficients were determined for specific days within a cycle, across different cycle months, for endogenous exposures to Pb, and following exogenous doses of Pb added directly to the menstrual fluid (Table 6).

**Table 6.** Partition coefficients describing Pb distribution in menstrual fluid.

Study purpose	Pb dose (ng/ml)	Donor	Cycle day	Cycle month	Menstrual RBC: menstrual plasma	Whole menstrual fluid: menstrual plasma
Day-to-day	10	RP54	1	–	73.5	20.6
			2		82.0	21.2
			3		11.2	3.0
			4		26.8	7.2
Month-to-month	10	RP54	2	1	107.4	32.9
				2	82.0	21.2
				3	93.4	18.6
	10	RP83	2	1	3.5	2.3
				2	1.3	1.1
				3	2.5	1.5
Dose response	0	RP54			63.7	17.0
	1		2	–	22.8	7.6
	10				94.3	24.2
	25				116.7	32.7
	0	RP83			6.9	3.6
	1		2	–	3.6	2.1
	10				2.4	1.6
	25				2.9	1.8
Donor-to-donor	10	RP54	2	–	94.3	24.2
		RP83			2.4	1.6
		RN1			3.0	1.5
		RN2			3.5	1.7
		RN4			4.4	1.7
		RN5			2.3	1.3
		RN6			1.6	1.2
		RN8			0.9	0.9
		Average (all donors)			14.1	4.3
		Average (without RP54)			2.6	1.4

Donor RP54 was utilized to evaluate *K*_M_rbc:M_plasma_ across the 4 d of a single menstrual cycle. The partition coefficients were relatively similar across these days, although variability was noted, with day 3 displaying the lowest partition coefficient among the 4 cycle days assessed. When analyzing the day 2 *K*_M_rbc:M_plasma_ across 3 different cycles, the coefficients differed by up to 4-fold when they were based on the endogenous concentration of Pb in the menstrual fluid. The average partition coefficient across these 3 mo was calculated to be 64 for RP54 and 6.9 for RP83. In scenarios where exogenous Pb (10 ng/ml) was dosed into the menstrual fluid over the same 3 mo, the average partition coefficient for RP54 increased to 94, whereas for RP83, it decreased to 2.4.

Further investigation into the effect of increasing concentrations of exogenous Pb (1, 10, or 25 ng/ml) revealed that *K*_M_rbc:M_plasma_ for RP54 and RP83 remained relatively consistent across the different doses. At a given dose, the partition coefficient was approximately 6 to 40 times lower for RP83 than for RP54. In addition to these findings, data from 6 additional donors were analyzed to determine their *K*_M_rbc:M_plasma_. When dosing with exogenous Pb (10 ng/ml), the average and standard deviation for *K*_M_rbc:M_plasma_ across all donors (*N* = 8) was 14.1 ± 34, and when RP54 was excluded, the value of *K*_M_rbc:M_plasma_ dropped to 2.6 ± 1.2. The *K*_M_fluid: M_plasma_ followed similar trends to *K*_M_rbc:M_plasma_, remaining relatively consistent across cycle days and cycle months for individual donors. The values for RP54 were approximately an order of magnitude higher than those for RP83. When considering all donors (*N* = 8), the average and standard deviation of *K*_M_fluid: M_plasma_ following exogenous Pb (10 ng/ml) exposure was calculated to be 4.3 ± 9.07, and when RP54 was excluded, the value was 1.4 ± 0.28.

### Binding of exogenous Pb in plasma isolated from menstrual fluid or systemic blood

To assess the free fraction of Pb within menstrual fluid, we determined the percentage of Pb that remained unbound following incubation with menstrual fluid-derived plasma from various cycle days and months, as well as with different doses of Pb. Additionally, we compared these results to the percent of Pb unbound in systemic blood-derived plasma obtained from a separate donor pool (Table 7).

**Table 7.** Percent of Pb unbound in plasma isolated from menstrual fluid and systemic blood.

Study purpose	Pb dose (ng/ml)	Donor	Cycle day	Cycle month	Percent unbound (average ± SD)^a^
Menstrual plasma day-to-day	10	RP54	1	–	1.5 ± 0.2
			2		2.0 ± 1.5
Menstrual plasma month-to-month	10	RP83	2	1	2.7 ± 0.2
				2	3.9^b^
				3	3.5 ± 0.8
Menstrual plasma dose response	1	RP54	2	–	3.6 ± 2.1
	10				2.0 ± 1.5
	25				1.2 ± 0.2
	1	RP83	2	–	10.3 ± 3.8
	10				3.3 ± 0.7^c^
	25				3.2 ± 0.2
Menstrual plasma donor pool	10	4-donor pool	2	–	12.2 ± 1.5
Systemic plasma^d^	1	N/A	N/A	N/A	21.4 ± 10.9
	10				6.3 ± 0.5
	25				7.5 ± 0.8

a Three replicates used unless indicated otherwise.

b Average of 2 replicates.

c Average of 8 replicates across 3 menstrual cycles.

d See methods for information on source of systemic plasma.

The percent of Pb unbound in menstrual fluid-derived plasma was consistent across different cycle days and cycle months when exogenous Pb (10 ng/ml) was added. The stability across menstrual days 1 and 2 suggests that the binding dynamics of Pb within menstrual fluid-derived plasma may not be significantly influenced by the timing within the menstrual cycle or by the month of collection. When evaluating the percent unbound in response to varying doses of exogenous Pb (1, 10, and 25 ng/ml), the average percent unbound for donor RP54 ranged from 1.2% to 3.6%, whereas for donor RP83, it ranged from 3.2% to 10%. When using a donor pool (*N* = 4) of menstrual fluid-derived plasma and dosing with exogenous Pb at 10 ng/ml, the average percent unbound was 12%.

### Mass-balance model describing the hypothetical release of Pb from a tampon and into menstrual fluid

A mass-balance model describing the movement of chemicals from a tampon into menstrual fluid and subsequent interactions within the vaginal environment was developed. The model was designed as a mechanistic framework to allow simultaneous integration of experimentally derived data describing chemical release, partitioning, reabsorption, and tissue permeation processes. The overall structure of the model is illustrated in Fig. 1. Model parameters were informed by available data for Pb, tampon materials, and vaginal physiology (Table 8), with a deliberate conservative bias.

**Table 8.** Parameters for mass-balance model describing Pb release from a tampon.

Name	Value	Unit	Description
R_release	66	ng/h	Pb release from tampon into menstrual fluid
Q_abs	1	ml/h	Menstrual fluid absorbed into tampon
Papp	1.0E−06	cm/s	Permeability through vaginal tissue
Area	21	cm^2^	Exposed vaginal tissues area
V_menses	0.5	ml	Free menstrual fluid volume
f_unbound	0.12	Unitless	Fraction of Pb unbound in menstrual fluid plasma
RBC_part_coef	2.6	Unitless	Menstrual fluid RBC: Plasma partition coefficient

Local sensitivity analyses were conducted to evaluate the influence of individual model parameters on predicted tissue uptake. Results are summarized in Table 9. The analysis indicated that multiple parameters have high sensitivity, reflecting their importance in governing model behavior. These findings support that the model structure captures the key processes relevant to chemical fate within the vaginal lumen and tissue interface.

**Table 9.** Sensitivity coefficients and designations.

Name	Sensitivity coefficient	Sensitivity designation
R_release	1.000	High
Papp	0.992	High
f_unbound	0.992	High
Area	0.992	High
Q_abs	−0.844	High
RBC_part_coef	−0.717	High
V_menses	−0.141	Low

The model was subsequently applied to simulate a representative tampon wear scenario of 4 h, with parameter values as listed in Table 8. During this period, Pb was assumed to be released continuously from the tampon at a rate of 66 ng/h into the menstrual fluid. Upon release, Pb is distributed between the plasma and RBC fractions of menstrual fluid based on experimentally derived partitioning. Within the plasma fraction, Pb was further distributed into bound and unbound fractions.

Pb present in menstrual fluid plasma (bound and unbound) and RBC-associated Pb was removed proportionally with menstrual fluid uptake into the tampon, modeled as a volumetric absorption rate of 1 ml/h, resulting in re-absorption of Pb back into the tampon. Only the unbound fraction of Pb in the menstrual plasma was assumed to be available for potential permeation across the vaginal tissue.

**Fig. 2.**
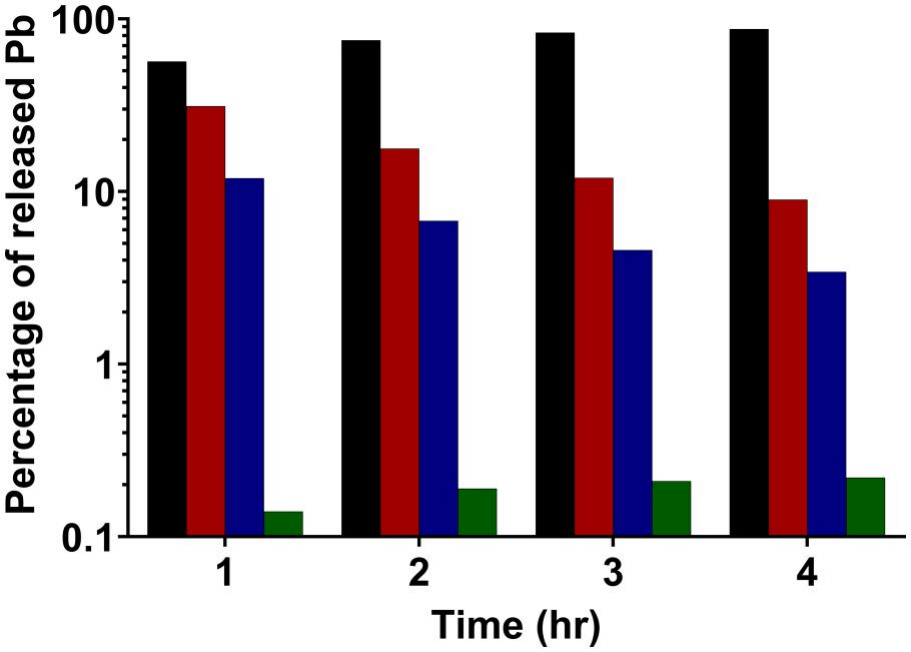
Simulation results from the mass-balance model describing the distribution of Pb that is hypothetically released from a tampon and into menstrual fluid. Parameters in the model were set to those listed in Table 8 and assume a release of Pb based on harsh, unrealistic extraction data. The model consists of 4 mass-balance compartments: Tampon reabsorption (black bars), menstrual fluid RBCs (red bars), menstrual fluid plasma (blue bars), vaginal tissue (green bars).

At the end of the 4-h wear time, the majority of released Pb was predicted to be reabsorbed into the tampon, accounting for 87% (231 ng Pb) of the total released mass (264 ng Pb). In contrast, a very small fraction of Pb was predicted to permeate into vaginal tissue, corresponding to 0.22% (0.58 ng Pb) of the released mass (Fig. 2). The remaining Pb mass was distributed within the free menstrual fluid, with 3.4% (9 ng Pb) present in the plasma fraction and 9% (24 ng Pb) associated with the RBC fraction.

**Fig. 3.**
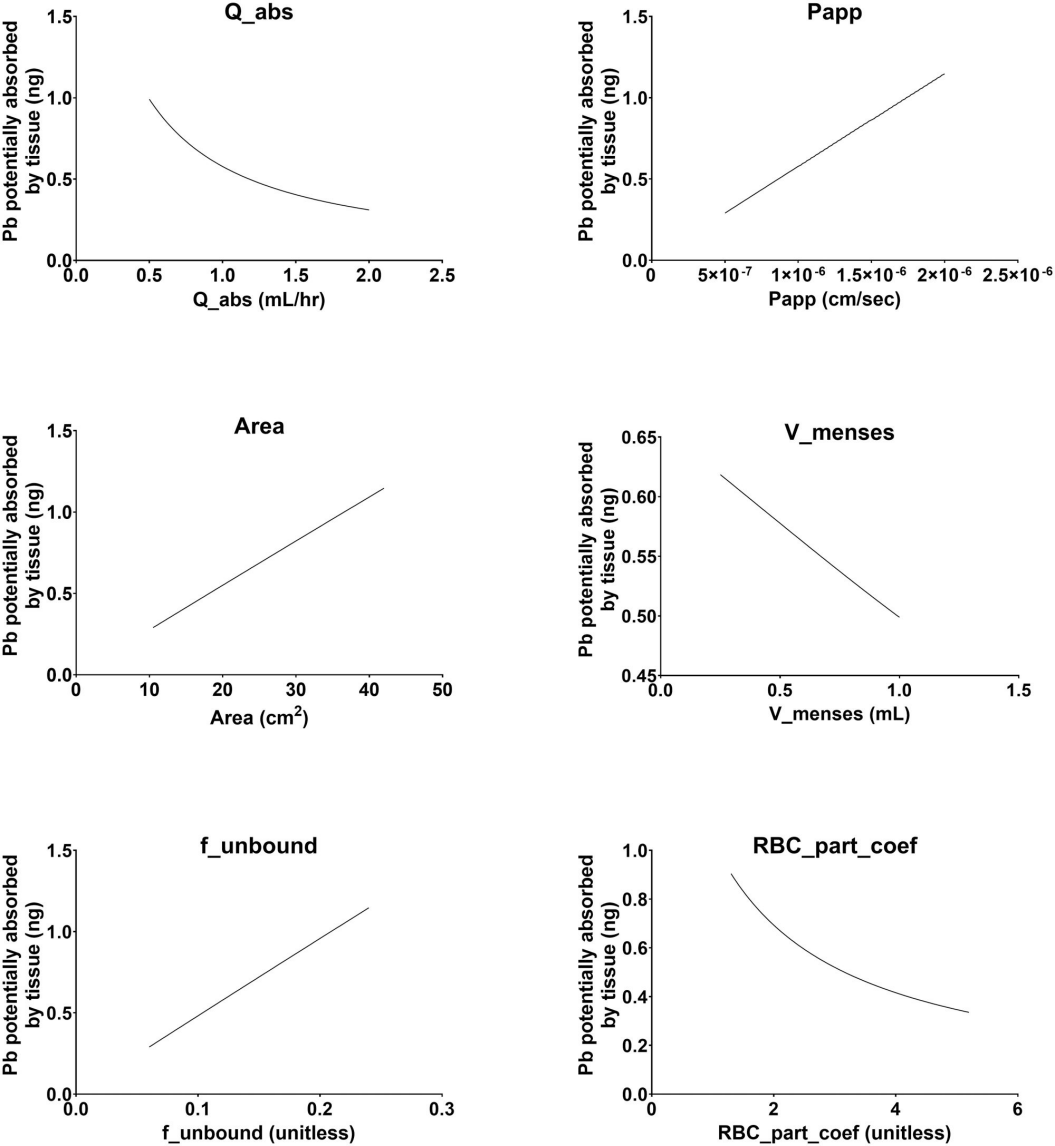
Individual model parameter values were varied 2-fold above and below their baseline values, and the resulting impact on tissue uptake of Pb was assessed.

To further evaluate model robustness, individual parameter values were varied 2-fold above and below their baseline values, and the resulting impact on tissue uptake of Pb was assessed (Fig. 3). Across all parameter perturbations evaluated, the maximum predicted amount of Pb that is potentially absorbed into vaginal tissue remained below 1.2 ng.

The model was further used to evaluate the impact of different Pb release dynamics from the tampon (Table 10). Under baseline conditions (simulation ID 1), Pb release was represented as a continuous, linear process occurring over the full 4-h wear period. Two additional simulations were conducted in which the time profile and extent of Pb release were modified. In simulation ID 2, all Pb present in the tampon was released into the menstrual fluid during the first 15 min of wear, whereas in simulation ID 3, only half of the Pb present in the tampon was released, with release likewise occurring during the first 15 min of wear. Despite these differences in release dynamics, simulations ID 2 and 3 resulted in potential tissue absorption fractions comparable to those predicted under baseline conditions. Across all scenarios, only a small fraction of released Pb was predicted to be potentially absorbed into vaginal tissue. At the end of the 4-h simulation period, the proportion of Pb reabsorbed into the tampon was greater in simulation ID 2 than in simulation ID 1. This difference reflects the continued release of Pb into the menstrual fluid throughout the simulation in the baseline scenario, whereas release in simulation ID 2 was confined to the initial 15-min interval. When Pb release in the baseline scenario was set to stop at 4 h, and the simulation was extended beyond the release period, the model predicted that 99.53% of the released Pb was reabsorbed into the tampon and 0.25% was potentially absorbed into vaginal tissue. In simulation ID 3, where only half of the Pb present in the tampon was released, the absolute mass of Pb absorbed into the tampon and potentially absorbed into the vaginal tissue was approximately half of that predicted in simulation ID 1. However, the relative distribution of Pb between reabsorption into the tampon and potential absorption into tissue remained consistent between simulations ID 1 and 3, indicating that the proportional fate of Pb is largely independent of the total mass released and the specific release timing under the conditions evaluated.

**Table 10.** Effects of Pb release rate and extent from tampons on potential tissue absorption over 4 h.

	Scenario parameters			Model results		
ID	Pb in tampon (ng)	Fraction of Pb released	Duration of Pb release (h)	Pb release from tampon (ng)	Pb re-absorbed into tampon (%)	Pb absorbed into tissue (%)
1	264	1	4	264	87.39	0.22
2	264	1	0.25	264	99.71	0.25
3	264	0.5	0.25	132	99.85	0.25

## Discussion

In the current work, we investigated the binding characteristics and distribution of Pb in menstrual fluid, exploring the implications of these findings for risk assessment related to chemical exposure from menstrual hygiene products. We conducted a series of experiments to measure endogenous concentrations of Pb, assessed the impact of exogenous Pb concentrations on its partitioning between plasma and RBC fractions of menstrual fluid, and evaluated the percent of Pb unbound in plasma isolated from menstrual fluid and systemic blood. We also developed a physiologically based mechanistic mass-balance model that describes the release of a chemical from a tampon into menstrual fluid and its subsequent fate within the vaginal lumen using the beyond worst-case presence results published previously (Shearston et al. 2024). If Pb is inadvertently present as a trace impurity in tampons used under typical conditions, it is predicted that less than 0.5% of the Pb in the tampon will be potentially absorbed into the vaginal tissue. If these parameters are altered to assume release of only ½ of the present Pb, the fraction predicted to be potentially absorbed into vaginal tissue remains low. Our findings highlight the binding properties of menstrual fluid and the behavior of a model contaminant within this biological matrix, providing crucial insights for understanding potential health risks.

Every subject in the study had a detectable endogenous concentration of Pb in their menstrual fluid, with the average concentration being 3 ppb (equivalent to 3 ng/ml or 0.3 µg/dl). The concentration of Pb in menstrual fluid aligns with systemic blood levels reported in the 2017 to 2018 National Health and Nutrition Examination Survey (NHANES), where the average Pb concentration for females was 0.66 µg/dl (6.6 ppb) and the 95th percentile reached 2.1 µg/dl (21 ppb) (CDC 2026). The CDC has established a blood lead reference value of 3.5 µg/dl (Ruckart et al. 2021), which the US FDA and other regulatory bodies utilize as the primary clinical “level of concern.” The current work demonstrated that the amount of Pb that may potentially be absorbed from a single tampon wear is on the order of ∼1 ng (equivalent to 0.001 µg), keeping in mind that this is based on a conservative assumption that all the Pb in a tampon would release from a tampon and into the menstrual fluid. Considering women use approximately 25 tampons per cycle (90th percentile) and have an average blood volume of 3.9 l (equivalent to 39 dl) (ICRP 2002), a conservative scenario where all of this potentially absorbed Pb instantaneously mixes with systemic blood would result in an approximate blood Pb concentration of 0.0006 µg/dl (0.025 µg Pb/39 dl blood). This concentration is significantly below the CDC blood lead reference value of 3.5 µg/dl. Furthermore, the concentration of Pb in menstrual fluid can also be used to estimate the total amount of Pb that is lost during menses. Multiplying the average concentration of Pb in menstrual fluid (3 ng/ml) by the average menstrual fluid volume per cycle (87 ml (Donoso et al. 2019)) indicates that approximately 261 ng of Pb will be excreted from the body each month via menses—an amount that is approximately an order of magnitude higher than the amount of Pb that may potentially be absorbed during tampon use.

The results of the sensitivity analysis for the mass balance model confirmed the importance of including absorption and distribution processes when trying to estimate the bioavailable dose of Pb that may result from a tampon exposure. In the current work, we measure the partitioning of Pb between the different fractions of menstrual fluid and determine the free fraction in plasma isolated from menstrual fluid. In the absence of permeability data for Pb across vaginal tissue, Caco-2 permeability data were utilized as a surrogate. This approach is considered appropriate for generating provisional model estimates. Further confidence in model predictions may be achieved by incorporating experimentally derived vaginal Pb permeability data. If future studies investigate vaginal absorption for Pb after tampon use, it would be crucial to use doses that accurately reflect realistic chemical concentrations encountered during normal product usage. Additionally, the choice of the absorption model is of paramount importance. Laboratory-grown models of vaginal tissue may not be considered adequate for measuring the absolute permeability of a chemical through vaginal tissue. Although these models may provide valuable insights into irritation (Machado et al. 2015, 2019) or facilitate relative comparisons between chemicals, they have not been extensively evaluated for predicting absorption in vivo. Instead, employing fresh, intact tissue would provide a more accurate representation of potential vaginal absorption. Ideally, the tissue should be sourced from humans or species with vaginal tissue that closely resembles the structure and function of the human vagina (McCracken et al. 2021).

The current work demonstrates the type of assays and data needed to clarify questions surrounding chemical distribution in menstrual fluid. By providing a detailed examination of Pb distribution and binding, we highlight the importance of considering these dynamics when estimating systemic exposure. Failing to include such information could lead to inaccurate assessments of chemical exposure and associated human health risks. The distribution of Pb observed in menstrual fluid in the present study was broadly comparable to that reported for systemic blood. In systemic circulation, Pb is predominantly associated with RBCs, with approximately 99% of Pb bound to the cellular fraction and only about 1% present in plasma (Sugawara et al. 1990). Consistent with this pattern, Pb in menstrual fluid was found to distribute preferentially to the RBC fraction; however, the magnitude of RBC partitioning was lower than that typically observed in systemic blood. One plausible explanation for this difference is the extensive hemolysis that occurs during menstruation, which results in the release of intracellular RBC components, including hemoglobin and aminolevulinic acid dehydratase, into the extracellular matrix of menstrual fluid. Pb is known to bind strongly to both hemoglobin and aminolevulinic acid dehydratase (Ong and Lee 1980; Bergdahl et al. 1997), and the presence of these proteins in the plasma fraction of menstrual fluid likely increases the capacity of this compartment to bind Pb, thereby reducing the apparent proportion of Pb associated with intact RBCs. In systemic blood, although only a small fraction of Pb is present in plasma, the majority of that plasma-associated Pb is protein-bound, with approximately 84% reported to be bound (DeSilva 1981, 1990; Schütz et al. 1996). In the present study, a similar extent of Pb binding was observed in plasma isolated from menstrual fluid, with approximately 88% of plasma-associated Pb present in the bound form. Together, these findings suggest that although the partitioning of Pb between cellular and plasma fractions differs somewhat between menstrual fluid and systemic blood, the underlying binding behavior of Pb to plasma components remains broadly consistent across these matrices. This similarity suggests that systemic blood partitioning and binding characteristics may provide valuable insights into those in menstrual fluid. Therefore, in the absence of specific menstrual fluid binding data, systemic blood data may serve as a pragmatic surrogate for preliminary estimates. Further investigation across more chemicals is warranted to ascertain the generalizability of this premise.

The modeling framework presented herein offers a structured approach for interpreting tampon-related analytical data within a physiologically and toxicologically relevant context. This framework may serve as a useful example for future assessments of other chemicals. Reapplication of this modeling approach to different chemicals would necessitate the use of chemical-specific data for menstrual fluid-derived partition coefficients and binding (RBCs and plasma), absorption across the vaginal tissue, and chemical release from the tampon. Furthermore, the framework can be extended beyond a deterministic model to incorporate a probabilistic approach. By utilizing distributions of parameter values as model inputs rather than point estimates, this approach would enable the prediction of exposure ranges.

In a regulatory toxicology context, standard solvent extraction testing for medical devices, such as tampons, serves a defined and important role within the broader framework for evaluating human safety. These analytical methods are designed to identify and quantify chemical constituents that may be present in a product; however, the detection of a chemical alone does not provide sufficient information to assess human health risk. Interpretation of such data requires consideration of exposure pathways, bioavailability, and relevant physiological processes. In some instances, datasets highlighting the presence of chemicals in medical devices or consumer products have been released without appropriate contextualization regarding exposure or toxicological relevance. When this occurs, the resulting interpretations may inadvertently overstate potential risk, contribute to public concern, and undermine trust in both regulatory processes and scientific assessment. Such outcomes may also lead consumers to seek alternative products that are not necessarily safer or better characterized. As demonstrated in the present work, only a very small fraction of a trace impurity present in a tampon is predicted to be potentially absorbed under realistic use conditions. Nonetheless, this distinction between chemical presence and biologically relevant exposure is frequently overlooked in discussions focused solely on analytical detection. A comprehensive safety assessment requires integration of product composition data with a mechanistic understanding of chemical release, partitioning, and absorption. We support continued efforts to characterize product composition and to evaluate the safety of both intentionally added ingredients and unavoidable trace impurities. However, reporting that emphasizes the mere presence of a “chemical” in a product without consideration of exposure magnitude or margins of safety can generate confusion rather than clarity.

## Authors contributions

Conceptualization: Corie Ellison, Kara Woeller. Methodology: Corie Ellison, Patrick Doyle, Christina Haven, Denise McClenathan, Cindy Obringer, Kara Woeller. Data curation: Corie Ellison, Patrick Doyle, Christina Haven, Denise McClenathan, Cindy Obringer, Kara Woeller. Data analysis: Corie Ellison, Patrick Doyle, Christina Haven, Denise McClenathan, Cindy Obringer, Kara Woeller. Writing: Corie Ellison, Patrick Doyle, Christina Haven, Denise McClenathan, Cindy Obringer, Kara Woeller.

## Supplementary Material

kfag052_Supplementary_Data
