# Supplementary material for: Integrating experimental data and mechanistic modeling to assess potential lead exposure from tampon use
Source: Toxicol Sci. 2026 May 7;209(5):kfag052. doi: 10.1093/toxsci/kfag052 (PMC13215380; doi:10.1093/toxsci/kfag052)
Supplement: kfag052_Supplementary_Data [file kfag052_supplementary_data.docx]

**Integrating experimental data and mechanistic modeling to assess potential lead exposure from tampon use**

Corie A. Ellison, Patrick R. Doyle, Christina A. Haven, Denise M. McClenathan, Cindy M. Obringer, Kara E. Woeller

**Berkley Madonna Model Code**

METHOD RK4

STARTTIME = 0

STOPTIME=4

DT = 0.02

;-------------------------------

; PARAMETERS

;-------------------------------

R_release_on = 66 ; ng/hr chemical release rate from tampon into menses while tampon is releasing

T_release_end = 4 ; hr duration of chemical release

R_release = IF TIME <= T_release_end THEN R_release_on ELSE 0 ; time-gated release

Q_abs = 1 ; mL/hr menstrual fluid absorbed into tampon

Papp = 1e-6 ; cm/sec permeability through vaginal tissue

Area = 21 ; cm2 exposed vaginal tissue area

V_menses = 0.50 ; mL free menstrual fluid volume

f_unbound = 0.12 ; fraction of plasma chemical that is free

RBC_part_coef = 2.6 ; RBC:plasma partition coefficient

sec_per_hr = 3600 ; seconds-to-hours conversion

;-------------------------------

; INITIAL CONDITIONS (ng)

;-------------------------------

INIT M_plasma = 0 ; chemical mass in menses plasma

INIT M_rbc = 0 ; chemical mass in menses RBCs

INIT M_tissue = 0 ; cumulative mass absorbed through tissue

INIT M_absorbed = 0 ; mass absorbed back into tampon with fluid

INIT M_released = 0 ; cumulative mass released from tampon

;-------------------------------

; PARTITIONING IN MENSES

;-------------------------------

Frac_plasma = 1 / (1 + RBC_part_coef) ; fraction of released mass in plasma

Frac_rbc = 1 - Frac_plasma ; fraction of released mass in RBCs

;-------------------------------

; CONCENTRATIONS (ng/mL)

;-------------------------------

C_plasma = M_plasma / V_menses ; plasma concentration in menses

C_rbc = M_rbc / V_menses ; RBC-associated concentration

C_free = f_unbound * C_plasma ; free plasma concentration

;-------------------------------

; FLUXES (ng/hr)

;-------------------------------

Absorb_plasma = Q_abs * C_plasma ; plasma chemical absorbed with fluid

Absorb_rbc = Q_abs * C_rbc ; RBC chemical absorbed with fluid

Tissue_flux = Papp * Area * C_free * sec_per_hr ; tissue uptake via permeability

;-------------------------------

; DIFFERENTIAL EQUATIONS

;-------------------------------

d/dt(M_plasma) =

R_release * Frac_plasma ; release apportioned to plasma

- Absorb_plasma ; loss via tampon fluid absorption

- Tissue_flux ; loss via tissue permeation

d/dt(M_rbc) =

R_release * Frac_rbc ; release apportioned to RBCs

- Absorb_rbc ; loss via tampon fluid absorption

d/dt(M_tissue) =

Tissue_flux ; accumulation in tissue

d/dt(M_absorbed) =

Absorb_plasma + Absorb_rbc ; mass returning to tampon

d/dt(M_released) =

R_release ; bookkeeping for mass balance

;-------------------------------

; MASS BALANCE DIAGNOSTICS

;-------------------------------

M_total = M_plasma + M_rbc + M_tissue + M_absorbed ; total mass accounted for

Mass_error = M_total - M_released ; should remain ~0
